# Epidemiology of Legionnaires’ Disease, Hong Kong, China, 2005−2015

**DOI:** 10.3201/eid2608.191244

**Published:** 2020-08

**Authors:** Yiu-Hong Leung, Chau-Kuen Lam, Yung-Yan Cheung, Chi-Wai Chan, Shuk-Kwan Chuang

**Affiliations:** Hong Kong Department of Health, Hong Kong, China

**Keywords:** Legionnaires’ disease, legionellosis, Legionella pneumophila, Legionella species, bacteria, epidemiology, surveillance, chronic medical conditions, environmental samples, Hong Kong, China

## Abstract

We reviewed findings of clinical, epidemiologic, and environmental investigations for 288 confirmed case-patients with Legionnaires’ disease reported in Hong Kong, China, during January 2005−December 2015. We found that chronic renal failure/impairment (adjusted odds ratio [aOR] 4.09), chronic pulmonary diseases (aOR 3.22), malignancy (aOR 3.04), and heart diseases (aOR 2.15) were independently associated with a higher risk for severe Legionnaires’ disease. However, patients with hyperlipidemia had a lower risk for severe outcome (aOR 0.17). *Legionella* positivity rate was 22% for 1,904 water samples collected. We found a higher positivity rate in summer months (28%−30%), which corroborated with months of highest rainfalls. Our novel finding that Legionnaires’ disease patients with hyperlipidemia had a lower risk for severe outcome deserves further study to confirm the observation and ascertain the underlying reason.

Legionnaires’ disease is caused by bacteria of the species *Legionella*, of which *Legionella pneumophila* serogroup 1 (Lp1) is the most virulent and the most common cause of disease (*1*). Legionnaires’ disease is transmitted mainly by inhalation of infectious aerosols; microaspiration of contaminated water is another possible mode of transmission (*2**,**3*).

Aerosol-generating systems, including cooling towers, whirlpools, decorative fountains, humidifiers or respiratory equipment, have been linked to Legionnaires’ disease cases and outbreaks (*4**,**5*). In particular, cooling towers are the most commonly reported source of infection for Legionnaires’ disease outbreaks, and hundreds of persons were affected in some outbreaks (*5**,**6*).

In Hong Kong, Legionnaires’ disease has been a notifiable infectious disease since 1994. Medical practitioners are required by law to report suspected or confirmed cases to the Centre for Health Protection (CHP) of the Department of Health. A confirmed case is defined as illness in a patient who had pneumonia and confirmatory laboratory results, including detection of Lp1 antigen in urine, detection of *Legionella* species nucleic acid or culture positive for *Legionella* species in respiratory specimens, or demonstration of a >4-fold increase in antibody titer against *L. pneumophila* in paired serum specimens. In this study, we reviewed the clinical, epidemiologic and environmental investigation findings for all confirmed Legionnaires’ disease cases reported during January 2005–December 2015.

## Materials and Methods

CHP conducted an epidemiologic investigation for all reported cases. We interviewed patients or their proxies and their attending doctors to obtain clinical and exposure history. We retrieved medical records of the patients to obtain supplementary clinical information, including complication and relevant microbiological and laboratory results.

In 2016, CHP adopted a risk-based strategy for environmental investigation and sampling for Legionnaires’ disease cases that environmental investigation and sampling from potential sources will be conducted only if certain criteria are met (e.g., definite nosocomial case). Before that time, environmental investigation and sampling were conducted for all case-patients except those who had not stayed in Hong Kong during the entire incubation period.

### Sample Collection

We conducted joint field visits with engineers of the Electrical and Mechanical Services Department to the patients’ residence. We collected water samples and environmental swab specimens from potential sources, including water outlets in kitchens and bathrooms of residence, and other aerosol generating system identified (e.g., humidifier or respiratory equipment). During field visits, we also looked for aerosol–generating systems, such as decorative fountains or fresh water cooling towers near patients’ residence. Water samples and environmental swab specimens would then be collected from these systems as appropriate. If patients had been exposed to other aerosol-generating systems in other places, such as workplace, club house, or recreational facilities, we would also conduct field visits to these places to collect water samples and environmental swab specimens.

Water samples were sent to the Public Health Laboratory Centre of CHP for testing of total *Legionella* count. Environmental swab specimens for detection of *Legionella* species were tested by culture. We performed molecular typing of *L. pneumophila* isolates from human and environmental samples by using pulsed-field gel electrophoresis and later by *Legionella* sequence-based typing according to the 7-gene protocol from the European Working Group for *Legionella* Infections sequence-based typing scheme (http://www.hpa-bioinformatics.org.uk/legionella/legionella_sbt/php/sbt_homepage.php).

### Data Collection

We reviewed case records of all confirmed Legionnaires’ disease cases in the study period. We retrieved information including sociodemographic characteristics (age, sex, ethnicity, smoking history, and occupation), medical history, clinical manifestations, and relevant laboratory and microbiological results from case records. We obtained population occupation data for the 2011 Hong Kong Population Census from the Census and Statistics Department.

### Data Analysis

We defined severe cases as those in patients who required admission to the intensive care unit for management of Legionnaires’ disease or in patients who died from this disease. Other cases were regarded as mild cases.

We entered all data into a spreadsheet by using Excel version 2010 (https://www.microsoft.com). For bivariate analysis, we computed crude odds ratio for sociodemographic and other variables that might predict severe illness. We used logistic regression for multivariate analysis. We used SPSS Statistics 14.0 (https://www.ibm.com) for all data analyses.

## Results

**Figure 1:**
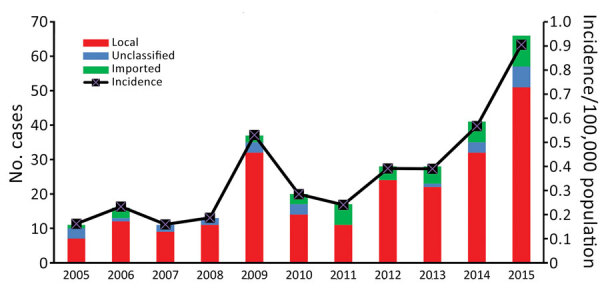
Annual number and incidence of Legionnaires' disease cases, Hong Kong, China, 2005–2015.

**Figure 2:**
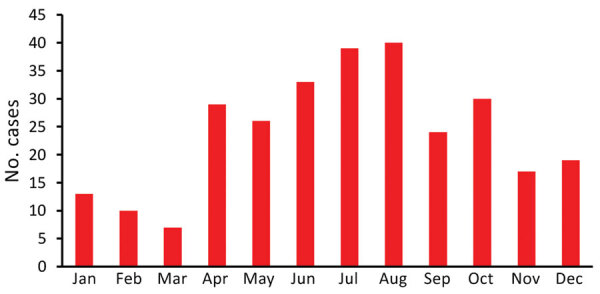
Number of Legionnaires' disease cases by month of onset, Hong Kong, China, 2005–2015. The onset date for 1 case was unknown.

A total of 288 confirmed Legionnaires’ disease cases were reported during the study period. The annual number of cases ranged from 11 to 66, and the annual incidence ranged from 0.16 cases/100,000 population to 0.91 cases/100,000 population (Figure 1). Cases with an onset during April–October accounted for 77% of all cases. However, more cases occurred during June–August (Figure 2).

### Sociodemographic Characteristics and Clinical Manifestations

Median age of the patients was 64 years (range 25–96 years, interquartile range 56–74 years), and 88% of case-patients were >50 years of age. A total of 86% of patients were male, 93% were Chinese, and 61% patients were either smokers or former smokers.

Information on occupation was unknown for 3 patients. A total of 164 patients (58%) were economically inactive (housewives, retired, or unemployed). Among the 121 working patients, 12% worked as drivers and 9% worked as security guards. The corresponding percentage of persons who worked in these occupations among the total working population in the 2011 Hong Kong Population Census was 4% for drivers and 3% for security guards.

Most (230, 80%) patients had a history of chronic medical illnesses. Hypertension (56%), diabetes (38%), and heart diseases (24%) were the most commonly reported illnesses (Table 1). All except 1 patient required hospital admission, and the duration of hospital stay ranged from 2 to 125 days (median 12 days). Fever (94%), cough (85%), and shortness of breath (65%) were the most commonly reported symptoms. Gastrointestinal symptoms were reported only by ≈20% of patients.

**Table 1:** Bivariate and multivariate analysis of factors associated with severe outcome of Legionnaires’ disease cases, Hong Kong, China, 2005–2015

Factor	Total, no. (%), n = 288	Severe, no. (%), n = 124	Mild, no. (%), n = 164	Crude odds ratio (95% CI)	Adjusted odds ratio (95% CI)
Male sex	248 (86)	108 (87)	140 (85)	1.16 (0.59–2.29)	1.43 (0.61–3.36)
Age >64 y	140 (49)	67 (54)	73 (45)	1.47 (0.92–2.34)	1.17 (0.66–2.05)
Smoker/former smoker	173 (61)*	79 (65)	94 (58)	1.38 (0.85–2.25)	1.55 (0.86–2.78)
History of chronic medical illnesses					
Chronic renal failure/impairment	46 (16)	30 (24)	16 (10)	2.95 (1.53–5.71)	4.09 (1.81–9.27)
Chronic pulmonary diseases	22 (8)	15 (12)	7 (4)	3.09 (1.22–7.82)	3.22 (1.10–9.42)
Malignancy	25 (9)	15 (12)	10 (6)	2.12 (0.92–4.89)	3.04 (1.17–7.91)
Heart diseases	69 (24)	40 (32)	29 (18)	2.22 (1.28–3.84)	2.15 (1.12–4.13)
Hyperlipidemia	53 (18)	13 (11)	40 (24)	0.36 (0.19–0.71)	0.17 (0.08–0.40)
Diabetes	109 (38)	49 (40)	60 (37)	1.13 (0.70–1.83)	0.94 (0.52–1.69)
Hypertension	161 (56)	77 (62)	84 (51)	1.56 (0.97–2.51)	1.62 (0.88–2.96)
Immunosuppression	22 (8)	10 (8)	12 (7)	1.11 (0.46–2.66)	0.75 (0.28–2.00)

*Information for 4 patients was unknown.

Respiratory failure developed in 119 (41%) patients, among whom 99 patients required intubation and mechanical ventilation. Deterioration of renal function developed in 149 (52%) patients, of whom 58 (39%) patients required renal replacement therapy. Other reported complications included septic shock (29%), acute coronary syndrome (6%), gastrointestinal bleeding (4%), and rhabdomyolysis (3%). A total of 114 (40%) patients required admission to an intensive care unit for management. A total of 42 patients died from Legionnaires’ disease. The case-fatality rate was 15%.

A total of 124 patients were classified as having severe cases and 164 patients as having mild cases. We showed by using bivariate analysis that patients with chronic renal failure/impairment, chronic pulmonary diseases, heart diseases, and hyperlipidemia had major associations with severe disease (Table 1). We showed by using multivariate analysis that patients with chronic renal failure/impairment (adjusted odds ratio [aOR] 4.09, 95% CI 1.81–9.27), chronic pulmonary diseases (aOR 3.22, 95% CI 1.10–9.42), malignancy (aOR 3.04, 95% CI 1.17–7.91), and heart diseases (aOR 2.15, 95% CI 1.12–4.13) were independently associated with a higher risk for severe disease. In contrast, patients who had hyperlipidemia had lower risk for severe outcome (aOR 0.17, 95% CI 0.08–0.40).

### Microbiological Investigation

**Figure 3:**
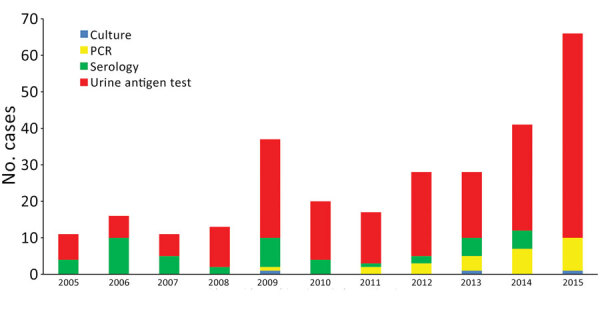
Number of Legionnaires' disease cases by confirmatory microbiological testing, Hong Kong, China, 2005–2015.

We provide the annual number of Legionnaires’ disease cases by confirmatory microbiological testing (Figure 3). Before 2008, ≈36%–63% of cases were confirmed by serologic analysis. Subsequently, most cases were confirmed by urine antigen test (UAT). An increasing number of cases since 2011 were also confirmed by PCR for respiratory specimens. Overall, 74% cases were confirmed by UAT, 16% by serologic analysis, and 9% by PCR. For the 26 case-patients given a diagnosis by PCR, 18 of them also had a UAT performed; only 2 of these patients had a positive result.

We analyzed respiratory specimens for *Legionella* culture for 166 (58%) patients and *L. pneumophila* was isolated for 71 (43%) patients. Among them, 69 had Lp1, 1 had *L. pneumophila* nonserogroup 1 (non-Lp1), and 1 had an unknown *L. pneumophila* serogroup. Among 16 case-patients given a diagnosis by PCR and negative UAT results, a respiratory specimen from 1 patient was culture positive for non-Lp1 and a specimen from another patient was positive for an unknown Lp serogroup. Another 2 patients had Lp1 nucleic acid detected by additional molecular testing.

### Environmental Investigations

Among the 288 cases, 256 had environmental investigations performed. A total of 1,904 water samples and 892 environmental swab specimens were collected from potential sources. The overall *Legionella* positivity rate for water samples was 22% (425/1,904), and it was generally higher for samples collected during June–August (28%–30%) (Table 2). Environmental investigations were conducted at 243 households; 85 (35%) had >1 water sample positive for *Legionella* species.

**Table 2:** *Legionella* culture results of water samples by month of collection, Hong Kong, China, 2005–2015

Month	No. water samples positive for *Legionella*/no. tested (%)
January	19/109 (17)
February	8/45 (18)
March	6/32 (19)
April	26/155 (17)
May	29/129 (23)
June	49/167 (29)
July	76/275 (28)
August	71/236 (30)
September	26/186 (14)
October	21/178 (12)
November	31/172 (18)
December	63/220 (29)*
Total	425/1,904 (22)

*For 1 case-patient, 39 of 43 water samples collected from patient’s workplace were positive for *Legionella* species. The percentage positive for December is 14% if this case-patient is excluded from analysis.

Regarding environmental swab specimens, 127 (14%) were positive for *Legionella* species. For collection sites, surface of water tap aerator, internal surface of water tap, shower head, or shower hose constituted ≈80% (101/127) of the positive samples. The positivity rate for water taps was 22% and that for showers was 6% (Table 3). In addition, we found that >30% of samples collected from water filters were positive for *Legionella* species. From the 240 households with environmental swab specimens collected, 56 (23%) had >1 environmental swab specimen positive for *Legionella* species.

**Table 3:** *Legionella* culture results of environmental swab specimens by site of collection, Hong Kong, China, 2005–2015

Site	No. water samples positive for *Legionella*/no. tested (%)
Water tap	74/332 (22)
Water tap aerator	47/248 (19)
Internal surface of water tap	27/84 (32)
Water filter	10/31 (32)
Shower*	27/459 (6)
Shower head	12/295 (4)
Shower hose	14/154 (9)
Water dispenser	2/17 (11)
Other	14/37 (28)
Total	127/892 (14)

*One sample was collected from shower head and shower hose.

### Source of Infection

We found that 78% (225) of case-patients contracted the infection locally, and 14% (39) cases were classified as imported cases (Figure 1). The source of infection of the remaining 8% of case-patients was regarded as unclassified because patients had stayed in Hong Kong and outside Hong Kong during the incubation period and had no exposure history for epidemiologic classification.

There were 3 nosocomial cases in patients who had stayed in a hospital for the entire incubation period. Disinfection of the relevant potable water system in the hospital by superheating and flushing and/or shock hyperchlorination was performed for all 3 case-patients. In addition, 11 case-patients were residents of long-term care facilities. Environmental investigations conducted failed to identify the source of infection for any of these case-patients.

All 288 patients had sporadic cases for which no outbreak was identified. Molecular typing was performed for 25 case-patients; only 1 case-patient had matching human and environmental samples. The patient was a 66 year-old retired man who had multiple illnesses. Lp1 was isolated from his tracheal aspirate and bronchoalveolar lavage; a water sample collected from his kitchen water tap, which had a filter; and a swab sample from the container of a respirator in his home. We found that the Lp1 isolates from the patient and the water samples had indistinguishable patterns by pulsed-field gel electrophoresis.

## Discussion

The incidence of Legionnaires’ disease in Hong Kong increased during the study period, showing a >4-fold increase from 0.16 cases/100,000 population during 2005 to 0.91 cases/100,000 population during 2015. A similar increasing trend has been observed in the United States and countries in Europe since the 2000s. In the United States, the incidence of legionellosis increased from 0.42 cases/100,000 population during 2000 to 1.62 cases/100,000 population during 2014 (*7*). In Europe, Legionnaires’ disease incidence increased from 0.54 cases/100,000 population during 2000 to 1.4 cases/100,000 population during 2015 (*8*,*9*).

The true incidence of Legionnaires’ disease was reported to be underestimated (*4**,**10*). The exact reason for the increasing incidence of Legionnaires’ disease is not known but is believed to be related to the increasing population of persons at high risk for infection, improved diagnosis and reporting, and increased use of UAT (*11*).

In Hong Kong, increasing incidence of Legionnaires’ disease during the study period might be related to increased use of more sensitive diagnostic tests. During 2005–2008, the average percentage of cases diagnosed by UAT was 50%, which increased to 78% during 2009–2015. The number of UATs performed showed an increase of 127% during 2015 compared with 2010; the annual increase was 11%–28%. The percentage positive results for UATs performed in the corresponding period decreased from 1.2% to 0.9%. In contrast, the number of reported Legionnaires’ disease cases increased from 20 during 2010 to 66 during 2015.

We also observed an increased number of cases diagnosed by PCR from 2011 onward. Among the 18 PCR-diagnosed cases for which a UAT was performed, only 2 showed positive UAT results, which implied that 16 of these PCR-diagnosed cases would not have been diagnosed if PCR had not been performed. With the development of commercially available multiplex PCR assays for respiratory pathogens, including *Legionella* species (*12*), it is expected that the Legionnaires’ disease incidence will continue to increase, and the percentage of Legionnaires’ disease cases diagnosed by PCR will also increase.

The clinical and epidemiologic characteristics of Legionnaires’ disease cases in Hong Kong were similar to those reported in other localities. Legionnaires’ disease incidence in Hong Kong showed an apparent seasonal trend with peak incidence during summer months. A similar situation has been reported for the United States, Europe, Canada, and Japan (*13*). Associations between legionellosis and several weather variables had been reported; the most consistent results are related to rainfall, and studies have identified small but major increases in risk for legionellosis with increased rainfall after a lag time of 1–2 weeks (*14*). In Hong Kong, ≈80% of rainfall occurred during May–September, and June–August had the greatest rainfall (*15*). Apart from Legionnaires’ disease incidence, our study also showed that water samples collected during June–August had the highest *Legionella* positivity rate.

Male sex, age >50 years, smoking, and a history of chronic diseases are well-established risk factors for acquiring Legionnaires’ disease (*4**,**10**,**13*). Our study showed consistent findings; 86% of patients were men and 88% of patients were >50 years of age. In addition, 61% of patients were either smokers or former smokers, and 80% had chronic medical illnesses.

In contrast, studies on the factors associated with severe outcome for Legionnaires’ disease are less common. Marston et al. reported that older age, male sex, nosocomial infection, immunosuppression, end-stage renal disease, and cancer were independently associated with death caused by Legionnaires’ disease (*16*). Chidiac et al. reported that older age and female sex were independent predictors of death for community-acquired legionellosis cases in France (*17*). Our findings demonstrated that age and sex of patients were not associated with severe outcomes, but a history of certain chronic medical illnesses was a major predictor of severe outcomes. We found that chronic renal failure/impairment (aOR 4.09), chronic pulmonary diseases (aOR 3.22), malignancy (aOR 3.04), and heart diseases (aOR 2.15) were independent risk factors for severe disease.

We also found that patients with hyperlipidemia had an 83% lower risk for severe outcomes than persons without this illness. This finding was unexpected. We postulate that this finding might be related to use of statins among patients with hyperlipidemia. In addition to its lipid-lowering effect, statins have been shown to attenuate acute lung injury by modulating neutrophil function, reducing proinflammatory cytokine release, and reducing vascular leak in experimental and animal studies; current evidence suggests that pretreatment with statins might have a beneficial effect in prevention of pneumonia and reducing severity of community-acquired pneumonia (*18*). We found that 66% (35/53) of patients with hyperlipidemia were documented to have used statins. Subgroup analysis showed that a lower percentage of persons who used statins had severe disease (23%, 8/35) than persons who did not use statins (28%, 5/18). However, these results were not significant, which might be caused by the small sample size. Additional studies should be conducted to confirm our hypothesis.

Delay in starting appropriate antimicrobial therapy could also be a risk factor for poor disease outcome. Unfortunately, our data did not capture the date of Legionnaires’ disease diagnosis and date of starting appropriate antimicrobial therapy, which is a limitation regarding the analysis of risk factors associated with severe outcome.

In our study, we found that patients who worked as drivers among working patients was overrepresented when compared with the general working population (12% vs. 4%). Several studies had reported being a professional driver as a risk factor for Legionnaires’ disease. Den Boer et al. found that being a professional driver was an independent risk factor for Legionnaires’ disease in a case–control study of sporadic community-acquired Legionnaires’ disease cases in the Netherlands during July 1998–June 2001 (*19*). A study on sporadic Legionnaires’ disease cases with onset during 2001–2006 in the United Kingdom also found that professional drivers had ≈5 times increased risk for Legionnaires’ disease (*20*).

The underlying reason for the association between working as a driver and Legionnaires’ disease is not known, but studies have reported that the air conditioning system, cabin air filter, or windshield washer fluid might be potential sources of transmission for *Legionella* species. Sakamoto et al. reported that a *Legionella* species was detected in 50% of the swab samples collected from the evaporator components of car air conditioning systems (*21*). Alexandropoulou et al. found that 32% of car cabin air filters tested were colonized with *L. pneumophila* (*22*). Another study reported that *Legionella* species was detected in 84% of windshield washer fluid samples collected from elementary school buses, and culturable cells were detected in aerosolized washer fluid during spraying of washer fluid (*23*).

Residential water supplies had been implicated as the source of infection for Legionnaires’ disease (*24*). Other studies have reported *Legionella* colonization in 6%–37% of residential potable water systems (*25**–**28*). Our study found that 35% of households had >1 water sample positive for *Legionella* species. Our finding was higher than that for another study in Hong Kong, which reported *Legionella* colonization in 22% of households surveyed (*29*). Apart from water samples, we also found that 23% of households had environmental swab specimens positive for *Legionella* species. Despite the high prevalence of households with *Legionella* colonization, matching *Legionella* isolates from human and environmental samples by molecular typing was found for only 1 case.

We also found that 32% of environmental swab specimens collected from water filters, 22% from water taps, and 6% from shower facilities were positive for *Legionella* species, but none of these samples were implicated as the source of infection for the cases in this investigation. Studies on the *Legionella* prevalence in swab samples have been reported rarely in the literature. A study from Japan reported that only 1 of 90 swab samples collected from 19 households were positive for *Legionella* species (*28*). The high prevalence of *Legionella* colonization in water fixtures deserves attention, and further studies are needed to delineate its relationship with Legionnaires’ disease in patients.

All cases of Legionnaires’ disease during the study period were sporadic, and no outbreak was detected in Hong Kong. The source of infection could not be determined for nearly all cases. However, this result might have been caused by the fact that only ≈10% of cases with environmental investigations conducted had positive isolates from human and environmental samples such that molecular typing could be performed because only 58% of case-patients had lower respiratory tract samples that could be tested for *Legionella* species. 

Some patients could not produce lower respiratory tract specimens, and in some instances, samples obtained were used only for bacterial culture. Communication and follow-up with the attending physicians and microbiology laboratories should be enhanced such that lower respiratory tract samples are collected for all Legionnaires’ disease patients to test for *Legionella* species to facilitate epidemiologic investigations.

In Hong Kong, Legionnaires’ disease incidence was increasing during the study period, but all cases were sporadic, and no outbreak was recorded. The apparent upward trend in incidence might be explained by increased use of more sensitive diagnostic tests. Legionnaires’ disease patients with chronic renal failure/impairment, chronic pulmonary diseases, malignancy, or heart diseases are at a higher risk for severe disease. Patients who had hyperlipidemia were found to have a lower risk for severe outcome, which is a novel finding that deserves further study to confirm the observation and ascertain the underlying reason. Environmental investigations showed that the *Legionella* positivity rate in water samples was higher in summer months, which corroborated with the seasonality of human infection and months that had greatest rainfall. Surveillance and epidemiologic investigation of Legionnaires’ disease cases is crucial in monitoring trends and other epidemiologic characteristics and for outbreak detection, which can contribute to formulation of prevention and control strategies for this disease.
